# HIV status, age at cervical Cancer screening and cervical cytology outcomes in an opportunistic screening setting in Nigeria: a 10-year Cross sectional data analysis

**DOI:** 10.1186/s13027-019-0263-4

**Published:** 2019-11-29

**Authors:** Jonah Musa, Chad J. Achenbach, Charlesnika T. Evans, Neil Jordan, Patrick H. Daru, Olugbenga Silas, Atiene S. Sagay, Rose Anorlu, Supriya D. Mehta, Firas Wehbe, Melissa A. Simon, Isaac F. Adewole, Lifang Hou, Robert L. Murphy

**Affiliations:** 1 Department of Obstetrics and Gynecology, College of Health Sciences, University of Jos, Jos, Plateau Nigeria; 2 Department of Preventive Medicine, Division of Cancer Epidemiology and Prevention, Feinberg School of Medicine, Northwestern University, Chicago, IL USA; 3 Institute of Global Health, Feinberg School of Medicine, Northwestern University, Chicago, IL USA; 4 Division of Infectious Diseases, Department of Medicine, Feinberg School of Medicine, Northwestern University, Chicago, IL USA; 5 Department of Preventive Medicine, Center for Health Care Studies, Institute for Public Health and Medicine, Feinberg School of Medicine, Northwestern University, Chicago, IL USA; 6 Center of Innovation for Complex Chronic Healthcare (CINCCH), Department of Veterans Affairs, Edward Hines Jr. VA Hospital, Hines, IL USA; 7 Department of Psychiatry & Behavioral Science, Feinberg School of Medicine, Northwestern University, Chicago, IL USA; 8 Department of Pathology, College of Health Sciences, University of Jos, Jos, Plateau State Nigeria; 9 Department of Obstetrics and Gynecology, College of Medicine, University of Lagos, Lagos, Lagos Nigeria; 10 Division of Epidemiology and Biostatistics, School of Public Health, University of Illinois at Chicago, Chicago, IL USA; 11 Department of Preventive Medicine, Division of Health and Biomedical Informatics, Feinberg School of Medicine, Northwestern University, Chicago, IL USA; 12 Department of Obstetrics and Gynecology, Preventive Medicine and Medical Social Sciences, Feinberg School of Medicine, Northwestern University, Chicago, IL USA; 13 Department of Obstetrics and Gynecology, College of Medicine, University of Ibadan, Ibadan, Oyo Nigeria; 14 Center for Population Epigenetics, Robert H. Lurie Comprehensive Cancer Center and Department of Preventive Medicine, Northwestern University Feinberg School of Medicine, Chicago, IL 60611 USA

**Keywords:** Cervical cancer screening, HIV status, Age at screening, Opportunistic screening, Cytology outcome, Nigeria

## Abstract

**Background:**

Invasive cervical cancer (ICC) is more prevalent in HIV infected women and occurs at younger median age than in HIV negative women. Organized cervical cancer screening (CCS) is presently lacking in Nigeria, and the age at CCS is not known in this population. We sought to examine the age at CCS, the cytology outcomes and whether outcomes differ by HIV infection status in an opportunistic screening setting.

**Methods:**

Cross-sectional analysis of data on a sample of women who had received a CCS in an opportunistic screening service in Jos, Nigeria over a 10-year time period (2006–2016). We used logistic regression models to estimate the independent effect of patient-reported HIV and age at CCS and odds ratios for abnormal cytology outcomes adjusting for other covariates. We also assessed the correlation between median age at CCS and severity of abnormal cervical cytology outcomes. Statistical analyses were done on STATA version 14, College Station, Texas, USA.

**Results:**

In a sample of 14,088, the median age at CCS was 37 years (IQR; 30–45). For HIV infected women vs. uninfected women, CCS occurred at earlier ages (35.0 ± 7.4 vs 38.2 ± 10.2 years, *p* < 0.001). All women, regardless of HIV status, who completed at least 7 or more years of education were 1.27 to 3.51 times more likely to have CCS before age 35 than women with less education. The predictors of an abnormal cervical cytology outcome at CCS were: age at CCS ≥ 35 (aOR = 3.57; 95% CI: 2.74, 4.64), multiparity ≥5 (aOR = 1.27; 95% CI: 1.03, 1.56), and provider-referral (aOR = 1.34; 95% CI: 1.09, 1.64). Irrespective of reported HIV status, we found a positive correlation between median age at CCS and severity of cytology outcome.

**Discussion:**

The age at CCS in women who have utilized cervical cancer screening in the study population is relatively late compared to the recommended age by most guidelines from developed settings. Late age at CCS correlates positively with severity of abnormal cytology outcome irrespective of HIV status. More educated women are more likely to have CCS at early age and less likely to have underlying abnormal cytology outcomes.

## Introduction

Even though invasive cervical cancer (ICC) is a preventable cancer, there are a half million new cases of ICC reported globally each year, with over 80% occurring in LMICs [1]. In Nigeria, over 53 million women are estimated to be at risk of ICC, and available cervical cancer screening (CCS) covers less than 9% of the population [2]. This lack of CCS availability contributes to approximately 14,000 new cases and 8000 deaths attributed to ICC every year [2]. The Global Burden of Cancer 2013 ranked ICC the 2nd most common in incidence and mortality for all cancers in Nigeria [3].

CCS is an important health care intervention for reducing ICC incidence and mortality with substantial benefits recorded in developed countries, where organized CCS programs are available [4–10]. In Nigeria the high prevalence of HIV [11] and the lack of organized CCS programs are substantial contributing factors to the high burden of ICC. In settings where organized CCS programs are lacking, the opportunity to have a screening test depends on several factors including the availability of a screening service and system support to overcome barriers to accessing such services; patient-related factors such as risk perception for ICC, illiteracy, and lack of awareness of CCS, or lack of knowledge and access to such screening [12–15]. Other important factors include cost of screening, health insurance coverage, education, perception of screening benefits and ability to overcome barriers to accessing services [13, 16].

Since the aim of CCS is to prevent cervical cancer through identification and treatment of precancerous cervical lesions, understanding the socio-demographic factors associated with abnormal cervical cytology outcomes could provide evidence for educating women and providers on the benefits of screening, particularly in women with certain characteristics. These predictors could also guide development of country-level screening guidelines for CCS and prevention. For instance, a French healthcare database on CCS provided evidence for not starting screening before age 25 [17], in comparison to the United States Preventive Service Task Force (USPSTF) guideline [18, 19] that recommends starting CCS at age 21.

Of particular interest are the findings from previous reports in sub-Saharan Africa that ICC is not only more prevalent in HIV infected women but occurs at a lower median age of 35 years compared to a median age of 40 years in women who are HIV negative [20]. Also, among women less than age 35, being HIV positive confers a 4-fold higher risk of having ICC compared to being HIV negative [20]. Therefore, HIV infected women may benefit from CCS by screening at relatively younger ages compared to HIV seronegative. Yet data from a large CCS program in Zambia, showed that the median age at first CSS was higher in HIV seropositive women compared to HIV seronegative women, reflecting that evidence related to HIV status, CCS, and ICC is not incorporated in the implementation of CCS [21].

In addition to the lack of an organized CCS program and poor coverage for available screening services, the age at CCS is not known in Nigeria [2]. We also do not know the effectiveness of screening in terms of the likelihood of detecting an underlying abnormal cervical cytology at the time of screening. In this paper, we sought to examine the age at CCS, the cytology outcomes and whether these outcomes differ by HIV status in an opportunistic screening setting in Nigeria.

## Methods

### Study design, setting and sample population

The detail of the study design, setting and sample derivation for this cross-sectional analysis has been described previously [22]. In brief, we utilized de-identified data of 14,088 women who had received a CCS at the “Operation Stop Cervical Cancer ‘(OSCC) Unit in, Jos, Nigeria, over a 10-year time period (2006–2016). We accessed the reported age at CCS and other relevant sociodemographic variables, risk factors, self-reported HIV status and the cytology outcomes reported by the cytopathologist. The cervical Pap cytology screening outcomes were reported according to the Bethesda 2001 cytology reporting system [23]. The primary independent variable for this analysis was self-reported HIV status at the time of CCS. The key dependent variables were age at CCS and the cytology outcome (see Additional file 1 for sample derivation and the dependent variables in the analysis of this manuscript). The description of the key variables and the cytology outcomes are provided in Additional file 2.

### Statistical analysis

#### Descriptive statistics

We performed summary statistics on continuous and categorical variables of the study sample and obtained means, medians and proportions for the independent and dependent variables. We also compared the baseline characteristics of the sample with the primary outcome. The Student’s t-test was used to assess differences in means of normally distributed continuous variables by HIV status. In this analysis, women who did not know their HIV status were treated as missing.

#### Analysis for age at CCS < 35 years as primary outcome

Since the principal exposure variable in this analysis was patient-reported HIV status, we estimated the mean age of women who received a CCS by patient-reported HIV status. We performed the Student’s t-test of differences in means between two groups (mean age of women who were “HIV infected” as group 1, and mean age of women who were HIV uninfected as group 2. Based on previous literature showing that the median age at developing ICC was 35 years in HIV infected women [20], we dichotomized the age at CCS in our sample at < 35 years and ≥ 35 years. We compared the baseline socio-demographic characteristics of the sample by age at CCS < 35 years compared to ≥35 years.

#### Bivariable and multivariable logistic regression model

To further understand the independent effect of patient-reported HIV on the age at CCS, we performed bivariable logistic regression analysis using various demographic variables as independent variables and dichotomizing the age at CCS as either < 35 years as the primary outcome “1” or age CCS ≥ 35 years as the referent category “0”. We also created dummy variables for other socio-demographic variables such as smoking, alcohol, years of completed education (< 7 years as group 1, 7–12 years as group 2 and > 12 years as group 3), history of ever been diagnosed with an STI, age at first coitus, history of vaginal infection, total lifetime number of sex partners, parity, and provider-referral. We first performed a bivariable logistic regression on each of these reported characteristics with age at CCS < 35 years as the primary outcome. We then used a multivariable logistic regression model to assess the independent predictive effect of patient-reported HIV on the likelihood of having a CCS at age < 35 years in our cervical cancer screening population. We used the backward selection method to build our final predictive model. We estimated 95% confidence intervals for each of these measures of association and corresponding *p*-values.

#### Analysis for abnormal cytology outcome at CCS as primary outcome

We estimated the relative proportions of the various categories of pap cytology outcomes at CCS reported according to the Bethesda system and the corresponding 95% CI. The median age at CCS for each of the cytology outcome categories and the corresponding interquartile range (IQR) were estimated. For analytic convenience and ease of interpretation we categorized the cytology report into three groups as follows: negative for intraepithelial lesion or malignancy (NILM) as category 1 (referent category); ASCUS and LSIL (mild cervical dysplasia) as category 2; and ASC-H, AGUS, HSIL, HSIL with suspicion for invasion (severe cervical dysplasia) as category 3. We also estimated the proportions for each of these sub-categories. We compared the baseline socio-demographic characteristics of the study sample by cervical cytology groups using the Pearson’s chi square or Fisher’s exact test where applicable and obtained corresponding *p*-values.

#### Bivariable logistic regression

We performed bivariable logistic regression to obtain the odds ratios of the association between baseline socio-demographic variables and abnormal cervical cytology, dummy variables were created for each of the cytology outcome categories with category 1 (NILM) as referent. We then performed separate bivariable logistic regression to estimate the likelihood of having mild cervical dysplasia (category 2) and severe cervical dysplasia (category 3) respectively at CCS for self-reported HIV and other socio-demographic characteristics in the study sample. For each of these categories, we estimated the unadjusted odds ratio, 95% CIs, and the corresponding *p*-values.

#### Multivariable logistic regression

We built a multivariable logistic regression model to assess the independent effect of self-reported HIV and other socio-demographic characteristics on the likelihood of an abnormal cervical cytology outcome report at CCS. As in the bivariable logistic regression model, we used category 1 cytology report (NILM) as referent. We then performed separate multivariable logistic regression models each for mild cervical dysplasia (category 2) and for severe cervical dysplasia (category 3). We used the backward selection method with *p* < 0.05 and the overall changes in the model effect to select the covariates that remain in each of the final predictive models. We estimated the 95% confidence intervals for each of these measures of association, and the corresponding *p*-values. The assessment of each model fit was by the Hosmer-Lemeshow goodnes’s-of-fit statistical test [24]. A p-value of greater than 0.05 was considered a good model-fit.

## Results

**Fig. 1:**
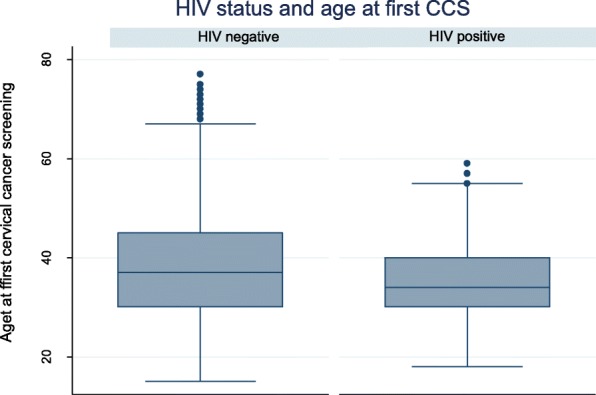
Box Plot of Age at CCS by patient-reported HIV status

During the study period 14,054 out of the 14,088 (99.8%) women reported the age at CCS, while 14,081 (99.95%) women had cervical cytology reports. The proportion of women who screened at < 35 years was significantly higher for women who were HIV infected (51.5%) compared to women who were HIV uninfected (40.2%) (*p* < 0.001). The mean age at CCS for HIV infected women was 35.0 ± 7.4 years compared to 38.2 ± 10.2 years for HIV uninfected women (*p*-value = 0.001). The Boxplot in Fig. 1 showed a significant difference in the age at CCS for HIV infected women compared to HIV uninfected. The results of the analyses for age at CCS have been summarized in Tables 1 and 2.

**Table 1:** Baseline socio-demographic characteristics by age at first CCS < 35 years versus ≥35 years in an opportunistic screening program in Jos, Nigeria (N = 14,051)

Variable	Age first CCS ≥ 35 years	Age first CCS < 35 years	p-value
HIV status			0.001^†^
Not infected	7870 (59.8)	5285 (40.2)	
Infected	341 (48.5)	362 (51.5)	
Age at first CCS (Mean ± SD)	8305 (44.5 ± 7.7)	5749 (22.7 ± 3.7)	0.001^‡^
No of Life-time sex			
partners(Mean ± SD)	6185 (2.2 ± 1.9)	5104 (2.2 ± 1.8)	0.503^‡^
Use of condom			
No	7307 (60.2)	4838 (39.8)	0.001^†^
Yes	404 (40.0)	605 (60.0)	
History of smoking			
No	8222 (59.3)	5653 (40.7)	0.272^†^
Yes	42 (53.2)	37 (46.8)	
History of Alcohol			
No	7625 (58.5)	5410 (41.5)	0.001^†^
Yes	635 (69.7)	276 (30.3)	
History of vaginal infection			
No	1536 (65.6)	805 (34.4)	0.001^†^
Yes	6517 (57.8)	4756 (42.2)	
Ever diagnosed with STI			
No	4963 (58.0)	3598 (42.0)	0.001^†^
Yes	744 (52.9)	662 (47.1)	
Age at first sex (Mean ± SD)	8193 (19.9 ± 4.1)	5651 (20.4 ± 3.8)	0.001^‡^
Education years completed (Mean ± SD)	6610 (11.8 ± 3.4)	5117 (11.9 ± 2.6)	0.062^‡^
Parity (Mean ± SD)	7818 (4.4 ± 2.5)	4317 (2.1 ± 1.7)	0.001^‡^

^*‡*^Student t-test and ^†^Pearson’s chi^2^. Percent in parenthesis*, SD* standard deviation

**Table 2:** Bivariable and multivariable Logistic regression model with unadjusted and adjusted odds ratio of the association between patient-reported HIV, other socio-demographic factors and the likelihood of first CCS at age < 35 years in an opportunistic cervical cancer screening program in Jos, Nigeria (*N* = 14,051)

Variable	OR (95% CI)	*P*-value	aOR (95% CI)	*P*-value
HIV status				
Not infected	1.0			
Infected	1.58 (1.36, 1.84)	0.001	1.18 (0.99, 1.41)	0.058
Referral group				
Self-referral	1.0	0.001	–	–
Provider-referral	0.75 (0.70, 0.80)			
Education (years completed)				
< 7 years	1.0			
7-12 years	3.12 (2.75, 3.53)	0.001	3.07 (2.69, 3.51)	0.001
> 12 years	1.53 (1.36, 1.72)	0.001	1.43 (1.27, 1.62)	0.001
Parity				
< 5	1.0			
≥ 5	0.51 (0.47, 0.55)	0.001	–	–
Age at first sex				
> 22 years	1.0			
≤ 22 years	0.83 (0.77, 0.90)	0.001	–	–
Total life-time sex partners				
< 3	1.0			
≥ 3	1.14 (1.05, 1.24)	0.001	–	–
Use of condoms during sex				
No	1.0			
Yes	2.26 (1.98, 2.58)	0.001	1.96 (1.70, 2.27)	0.001
History of vaginal infection				
No	1.0			
Yes	1.39 (1.27, 1.53)	0.001	1.29 (1.15, 1.43)	0.001
Ever diagnosed with STIs				
No	1.0			
Yes	1.23 (1.10, 1.37)	0.001	–	–
History of Smoking (*N* = 13,954)				
No	1.0			
Yes	1.28 (0.82, 2.0)	0.273	1.63 (0.93, 2.83)	0.086
Alcohol consumption (13,946)				
No	1.0			
Yes	0.61 (0.53, 0.71)	0.001	–	–

*The Hosmer-Lemeshow goodness-of-fit p-value = 0.538, Pseudo R*^*2*^ *= 0.0363, LR (chi*^*2*^*) = 521.35*

**Fig. 2:**
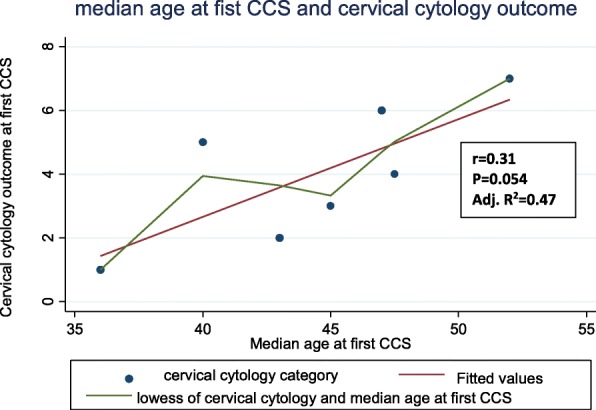
Scatter plot of the median age at CCS and the cervical cytology outcome (1 = NILM, 2 = ASCUS, 3 = LSIL, 4 = ASCUS-H, 5 = AGUS, 6 = HSIL and 7 = HSIL with suspicion for invasion)

The baseline sociodemographic and cytology outcomes of the study sample has been published in an earlier related report and shown in Table 3 [22]. In brief, 85.7% of the study sample had NILM, while 9.7 and 4.6% had mild and severe cervical dysplasia respectively. Specifically, 4.1% (95% CI: 3.8, 4.5%) with ASCUS, 5.6% (95% CI: 5.3, 6.0) with LSIL, 1.6% (95% CI: 1.4, 1.8) with ASC-H, 0.2% (95% CI: 0.2, 0.3) with AGUS, 2.5% (95% CI: 2.3, 2.8) with HSIL, and 0.2% (95% CI, 0.2, 0.3) with HSIL with suspicion for invasion. The median age for the various cytology categories were: 36 years (IQR; 30–43) for NILM, 43 years (IQR; 36–50) for ASCUS, 45 years (IQR; 35–52) for LSIL, 47.5 years (IQR; 38–55) for ASCUS-H, 40 years (95% CI, 34–52) for AGUS, 47 years (IQR; 39–55) for HSIL, and 52 years (IQR; 43–60) for HSIL with suspicion for invasion. The scatter plot in Fig. 2 of the median age at CCS and the predicted cytology outcome category reflects a positive linear relationship between median age and severity of cytology outcome at cervical screening (r = 0.31; Adj. R^2^ = 0.47; *p*-value = 0.054). Self-reported HIV status was not significantly associated with mild or severe cervical dysplasia in the study sample (p-value = 0.930). The association between other socio-demographic variables with cervical cytology outcomes are displayed in Table 4.

**Table 3:** Summary statistics of the socio-demographic and cytology outcomes of women who received first CCS in an opportunistic cervical cancer screening program in Jos Nigeria (*N* = 14,088)

Characteristics	Descriptive statistics (Mean ± SD, Median, IQR or % in parentheses)	95% Confidence intervals
Age at CCS	37; IQR, 30–45	
Age groups at CCS		
< 21 years	1.1	1.0, 1.3
21–30	24.7	24.0, 25.4
31–40	37.3	36.5, 38.1
41–50	25.4	24.6, 26.1
51–60	8.9	8.5, 9.4
61–70	2.1	1.8, 2.3
≥ 71	0.2	0.2, 0.3
Missing	0.2	0.2, 0.3
Age at first sex	20; IQR, 18–22	
Education years completed	13; IQR, 12–14	
Annual household income in USD	3300; IQR, 1920-4800	
HIV status		
Infected	703 (5.0)	4.6–5.5
Not infected	13,155 (93.4)	93.0–93.8
Unknown (missing)	230 (1.6)	1.4–1.9
History of Vaginal infection		
Yes	80.0	79.4–80.7
No	16.6	16.0–17.2
Missing	3.4	3.1–3.7
Use of condoms		
Yes	7.4	6.8–7.6
No	86.2	85.6–86.8
Missing	6.6	6.2–7.1
Ever diagnosed with an STI		
Yes	10.0	9.5–10.5
No	60.8	60.0–61.6
Missing	29.3	28.5–30.0
Types of STIs		
Gonorrhea	17.0	14.0–20.5
Trichomonads	6.7	4.8–9.2
Hepatitis	40.5	36.4–44.8
Chlamydia	28.7	17.3–47.1
HPV/Genital warts	5.9	4.2–8.3
Syphilis	4.8	3.3–7.0
Herpes	3.4	2.2–5.4
PID/Unspecified	18.3	15.6–22.3
# of Lifetime sex partners	2; IQR, 1–3	
Parity	3; IQR, 2–3	
History of smoking		
Yes	0.6	0.5–0.7
No	98.5	98.3–98.7
Missing	1.0	0.8–1.1
History of Alcohol		
Yes	6.5	6.1–6.9
No	92.5	92.1–93.0
missing	1.0	0.9–1.2
Race		
Black	99.7	99.6–99.8
Others	0.1	0.1–0.2
Missing	0.2	0.1–0.30
Cytology outcome at CCS		
NILM	85.7	85.1–86.3
ASCUS	4.1	3.8–4.5
LSIL	5.6	5.3–6.0
ASC-H	1.6	1.4–1.8
AGUS	0.2	0.2–0.3
HSIL	2.5	2.3–2.8
HSIL, suspicion for invasion	0.2	0.2–0.3
Cytology category at CCS		
Normal cervical cytology	85.7	85.1–86.3
Mild cervical dysplasia	9.7	9.3–10.2
Severe cervical dysplasia	4.6	4.2–4.9

*SD* standard deviation*, IQR* Interquartile range, % (Percent)

**Table 4:** Baseline socio-demographic characteristics by cervical cytology category at CCS in an opportunistic screening program in Jos, Nigeria (*N* = 14,081)

Variable	NILM	Mild Dysplasia	Severe dysplasa	*p-*value
HIV status				
Not infected	11,261 (85.7)	1288 (9.8)	599 (4.6)	0.930^a^
Infected	605 (86.1)	68 (9.7)	30 (4.3)	
Age at CCS				
< 35 years	5367 (93.4)	288 (5.0)	94 (1.6)	0.001^***†***^
≥ 35 years	6701 (80.4)	1083 (13.0)	548 (6.6)	
Total # lifetime sex partners				
< 3	6727 (85.5)	763 (9.7)	374 (4.8)	0.001^a^
≥ 3	3035 (88.7)	271 (7.9)	114 (3.3)	
Use of condom				
No	10,436 (86.0)	1166 (9.6)	540 (4.4)	0.002^a^
Yes	904 (89.5)	81 (8.0)	25 (2.5)	
History of smoking				
No	11,899 (85.8)	1340 (9.7)	630 (4.5)	0.145^b^
Yes	63 (79.8)	13 (16.4)	3 (3.8)	
History of Alcohol				
No	11,212 (86.1)	1230 (9.4)	588 (4.5)	0.001^a^
Yes	743 (81.7)	123 (13.5)	44 (4.8)	
History of vaginal infection				
No	1919 (82.0)	276 (11.8)	145 (6.2)	0.001^a^
Yes	9752 (86.6)	1036 (9.2)	480 (4.2)	
Ever diagnosed with STI				
No	7431 (86.8)	763 (8.9)	365 (4.3)	0.843^a^
Yes	1228 (87.3)	122 (8.7)	56 (4.0)	
Age at first sex				
≥ 22 years	8025 (84.5)	996 (10.5)	476 (5.0)	0.001^a^
< 22 years	3843 (88.5)	345 (8.0)	153 (3.5)	
Education years completed				
< 7 years	1366 (83.8)	172 (10.5)	93 (5.7)	0.001^a^
7–12 years	3078 (89.8)	256 (7.5)	93 (2.7)	
> 12 years	5834 (87.6)	584 (8.8)	244 (3.6)	
Parity				
< 5	7382 (88.2)	699 (8.4)	288 (3.4)	0.001^a^
≥ 5	2894 (77.0)	563 (14.9)	303 (8.1)	

^a^Pearson’s chi^2^. ^b^Fisher’s Exact. Percent in parenthesis

### Unadjusted and adjusted logistic regression model of self-reported HIV and other sociodemographic variables and mild cervical dysplasia

In the unadjusted regression model, self-reported HIV infection was not significantly associated with mild cervical dysplasia (OR = 0.99; 95% CI: 0.77, 1.28). The sociodemographic factors that were significantly associated with mild cervical dysplasia were: age at CCS ≥35 years (OR = 2.83; 95% CI: 2.48, 3.24), multiparity ≥5 (OR = 1.46; 95% CI: 1.31, 1.64), age at first sexual intercourse ≤22 years (OR = 1.23; 95% CI: 1.08, 1.41), provider-referral (OR = 1.88; 95% CI: 1.67, 2.11), history of ever smoked cigarettes (OR = 1.84; 95% CI: 1.01, 3.35) and history of alcohol consumption (OR = 1.50; 95% CI: 1.23, 1.83). One notable finding in the unadjusted model is that women with 7 or more completed years of education were significantly less likely to have mild cervical dysplasia at CCS than women with less than 7 completed years of education (7–12 years, OR = 0.68; 95% CI: 0.56, 0.84; > 12 years, OR = 0.82; 95% CI: 0.68, 0.96). These unadjusted ORs are presented in Table 5.

**Table 5:** Bivariable and multivariable Logistic regression with unadjusted and adjusted odds ratio of the association of patient-reported HIV and other sociodemographic variables and mild cervical dysplasia at CCS in Jos, Nigeria (N = 13,554)

Variable	OR (95% CI)	*P*-value	aOR (95% CI)	*P*-value
HIV status				
Uninfected	1.0			
Infected	0.99 (0.77, 1.28)	0.953	1.04 (0.80, 1.36)	0.747
Age at CCS				
< 35 years	1.0			
≥ 35 years	2.83 (2.48, 3.24)	0.001	2.56 (2.23, 2.95)	0.001
Referral group				
Self-referral	1.0			
Provider-referral	1.88 (1.67, 2.11)	0.001	1.75 (1.56, 1.98)	0.001
Education (years completed)				
< 7 years	1.0			
7-12 years	0.68 (0.56, 0.84)	0.001	–	–
> 12 years	0.82 (0.68, 0.96)	0.025	–	–
Parity				
< 5	1.0			
≥ 5	1.46 (1.31, 1.64)	0.001	1.21 (1.08, 1.36)	0.001
Age at first sex				
> 22 years	1.0			
≤ 22 years	1.23 (1.08, 1.41)	0.002	–	–
Total life-time sex partners				
< 3	1.0			
≥ 3	0.80 (0.69, 0.93)	0.003	–	–
Use of condoms during sex				
No	1.0			
Yes	0.82 (0.65, 1.04)	0.103	–	–
History of vaginal infection				
No	1.0			
Yes	0.76 (0.69, 0.87)	0.001	0.81 (0.70, 0.94)	0.004
Ever diagnosed with STIs				
No	1.0			
Yes	0.97 (0.79, 1.19)	0.772	–	–
History of Smoking				
No	1.0			
Yes	1.84 (1.01, 3.35)	0.045	–	–
Alcohol consumption				
No	1.0			
Yes	1.50 (1.23, 1.83)	0.001	1.38 (1.13, 1.70)	0.002

Hosmer-Lemeshow Goodnes-of-fit *p*-value = 0.145, LR (chi2) = 365.90, Pseudo R^2^ = 0.0425

In the adjusted logistic regression model including age at CCS ≥ 35, provider-referral, multiparity ≥5, history of vaginal infection and alcohol consumption, the effect of self-reported HIV infection was not significantly associated with mild cervical dysplasia (aOR = 1.04; 95% CI: 0.80, 1.36). The sociodemographic variables that were independently associated with mild cervical dysplasia were: age at CCS ≥ 35 (aOR = 2.56; 95% CI: 2.23, 2.95), multiparity ≥5 (aOR = 1.21; 95% CI: 1.08, 1.36), provider-referral (aOR = 1.75; 95% CI: 1.56, 1.98) and history of alcohol consumption (aOR = 1.38; 95% CI: 1.38; 95% CI: 1.13, 1.70). These adjusted ORs are presented in Table 5.

### Unadjusted and adjusted logistic regression model of self-reported HIV and other sociodemographic variables and severe cervical dysplasia

In the unadjusted regression model, self-reported HIV infection was not significantly associated with severe cervical dysplasia (OR = 0.93; 95% CI: 0.64, 1.35). The sociodemographic factors that were significantly associated with severe cervical dysplasia were: age at CCS ≥ 35 years (OR = 4.24; 95% CI: 3.40, 5.29), multiparity ≥5 (OR = 1.85; 95% CI: 1.58, 2.17), age at first sexual intercourse ≤22 years (OR = 1.32; 95% CI: 1.08, 1.60), provider-referral (OR = 1.27; 95% CI: 1.08, 1.49). Similar to the unadjusted model for mild dysplasia, women with 7–12 completed years or more of education were significantly less likely to have severe cervical dysplasia at CCS than women with less than 7 completed years of education (7–12 years, OR = 0.46; 95% CI: 0.34, 0.62; > 12 years, OR = 0.63; 95% CI: 0.49, 0.80). The unadjusted ORs are presented in Table 6.

**Table 6:** Bivariable and multivariable Logistic regression with unadjusted and adjusted odds ratio of the association of patient-reported HIV and other sociodemographic variables and severe cervical dysplasia at CCS in Jos, Nigeria (*N* = 11,345)

Variable	OR (95% CI)	*P*-value	aOR (95% CI)	*P*-value
HIV status				
Uninfected	1.0			
Infected	0.93 (0.64, 1.35)	0.704	1.26 (0.83, 1.92)	0.276
Age at first CCS				
< 35 years	1.0			
≥ 35 years	4.24 (3.40, 5.29)	0.001	3.57 (2.74, 4.64)	0.001
Referral group				
Self-referral	1.0			
Provider-referral	1.27 (1.08, 1.49)	0.004	1.34 (1.09, 1.64)	0.005
Education (years completed)				
< 7 years	1.0			
7-12 years	0.46 (0.34, 0.62)	0.001	0.65 (0.48, 0.88)	0.006
> 12 years	0.63 (0.49, 0.80)	0.001	0.75 (0.58, 0.98)	0.034
Parity				
< 5	1.0			
≥5	1.85 (1.58, 2.17)	0.001	1.27 (1.03, 1.56)	0.025
Age at first sex				
> 22 years	1.0			
≤22 years	1.32 (1.08, 1.60)	0.006	–	–
Total lifetime sex partners				
< 3	1.0			
≥3	0.69 (0.56, 0.86)	0.001	–	–
Use of condoms during sex				
No	1.0			
Yes	0.55 (0.36, 0.82)	0.004	–	–
History of vaginal infection				
No	1.0			
Yes	0.67 (0.56, 0.82)	0.001	0.67 (0.53, 0.84)	0.001
Ever diagnosed with STIs				
No	1.0			
Yes	0.93 (0.70, 1.24)	0.627	–	–
History of Smoking				
No	1.0			
Yes	0.83 (0.26, 2.64)	0.751	–	–
Alcohol consumption				
No	1.0			
Yes	1.08 (0.79, 1.47)	0.651	–	–

Hosmer-Lemeshow Goodnesss-of-fit *p*-value 0.798. LR (chi^2^)-178.15, Pseudo R^2^ = 0.0497

In the adjusted logistic regression model including age at CCS ≥ 35, provider-referral, multiparity ≥5, history of vaginal infection, 7–12 years of completed education, and > 12 years of completed education, the effect of self-reported HIV infection was not significantly associated with severe cervical dysplasia (aOR = 1.26; 95% CI: 0.83, 1.92). The sociodemographic variables that were independently associated with severe cervical dysplasia were: age at CCS ≥ 35 (aOR = 3.57; 95% CI: 2.74, 4.64), multiparity ≥5 (aOR = 1.27; 95% CI: 1.03, 1.56), and provider-referral (aOR = 1.34; 95% CI: 1.09, 1.64). Women with 7–12 completed years of education (aOR = 0.65; 95% CI: 0.48, 0.88), > 12 completed years of education (aOR = 0.75; 95% CI: 0.58, 0.98), and history of vaginal infection (aOR = 0.67; 95% CI: 0.53, 0.84) were significantly less likely to have severe cervical dysplasia at first CCS. These adjusted ORs are presented in Table 6.

## Discussion

The results of our analyses have contributed to our understanding of socio-demographic factors associated with utilization and cytology screening outcomes in an opportunistic CCS program in Jos, Nigeria. We found that women who had utilized the opportunistic CCS service in the population had screening at a median age of 37 years (IQR 30–45). We also found that on average, women who were HIV infected had CCS at a younger age than women who were HIV uninfected. Also, women who completed at least 7 years of education were 1.27 to 3.51 times more likely to have had CCS before age 35 than women with less education.

Our study findings have significant implications for cervical cancer prevention and screening in Nigeria. The median age at CCS is relatively late at 37 years, and this is of concern for cervical cancer prevention and control given the evidence that ICC occurs at a median age of 35 years in HIV infected women, 40 years in HIV uninfected women, and 38 years in women with unknown HIV status [20]. The relatively late screening age in our sample suggests that many women may have already developed precancerous conditions of the cervix or invasive cancer at the time of CCS. This finding could also explain the high rate of advanced stage ICC with high death rates as reported in previous studies [25–28].

Related to the age at CCS, an earlier study report from a district hospital in Abuja, Nigeria’s federal capital, found a mean age of 32.0 ± 6.6 years at first CCS by visual inspection with acetic acid (VIA) [29]. Compared to the mean age of 35.0 ± 7.4 years at first CCS in our study sample, the slightly lower mean age at first screening in the Abuja HIV population could partly be explained by the mode of screening using VIA, and the specific program intervention, which involved active interaction between HIV infected women receiving antiretroviral therapy and provider-initiated CCS with VIA during the intervention period [29]. VIA is technically less sophisticated than cytology-based screening which is usually done in tertiary health care facilities with cytopathologic laboratory support. Moreover, cytology-based screening methods have been shown to be more specific in detecting cervical precancer in HIV infected populations irrespective of immune status and antiretroviral treatment [30]. Overall, the findings on age at screening in our study population have broadened our knowledge and understanding of the current situation on CCS services in Nigeria and the need to leverage these data for health policy advocacy at state’s and federal ministries of health to guide prevention efforts particularly the availability and access to screening either by cytology-based or “see-and-treat” by VIA as recommended by WHO for early detection and treatment of cervical precancerous conditions.

We also analyzed the association between self-reported HIV and abnormal cervical cytology outcome at CCS in our study sample. We found that self-reported HIV was not significantly associated with having either mild or severe cervical dysplasia at the time of first CCS. The weak association between HIV and abnormal cervical cytology outcome could be partly explained by the wide-spread use of highly active antiretroviral therapy (HAART) in our study sample. Also, this was a cross-sectional data analysis with no follow up element to ascertain the risk of incident cervical abnormalities in HIV infected compared to women who were HIV negative. However, follow up data in a US population did not find a significant difference in incident cervical dysplasia and cancer in HIV population on successful HAART [31, 32]. However, we found that women who had CCS at age ≥ 35 years were 2.6 and 3.6 times more likely to have an underlying mild and severe cervical dysplasia, respectively. The utility of HSIL for early detection of cervical cancer has been studied in older women and its sensitivity for cancer was 89% in women screened at age 40–69 and 83% in women screened at age ≥ 70 years [33]Therefore, our study findings showing a severe dysplasia rate of 4.6% and that older age is a significant predictor of underlying severe dysplasia are useful findings that could contribute to developing and implementing CCS policy and guidelines with respect to age at which to start CCS in Nigeria.

A closer assessment of the relationship between age at CCS and abnormal cervical cytology outcome showed a positive correlation between median age at CCS and the severity of underlying cervical cytologic abnormality (Fig. 2). Though the strength of this correlation is modest with a borderline statistical significance (r = 0.31; *p* = 0.054 and adjusted R^2^ = 0.47), the median age at diagnosis of these abnormalities and the corresponding interquartile range suggest that implementing a Nigerian CCS policy and guidelines that cover screening between age 30 and 60 years may be an effective screening recommendation. Although our data are limited to one federal academic tertiary medical institution in northern Nigeria, a subsequent cost-effectiveness analysis can characterize and add to understanding the value of extending CCS outside this age range in the Nigerian population. Such understanding is crucial in resource-constrained settings where health insurance coverage is limited. If subsequent cost-effectiveness analysis support screening within this age range, health policy makers could implement health insurance coverage for CCS for women ages 30 to 60 in Nigeria. However, there is need to obtain more large-scale screening data across the country to increase the precision of these estimates.

Our analyses also found that multiparity ≥5 was significantly associated with mild or severe cervical dysplasia at first CCS. Specifically, women with parity ≥5 were 1.85 and 1.27 times more likely to have an underlying mild or severe cervical dysplasia, respectively, at the time of first CCS. Studies on the cofactors in cervical pre-cancer and progression to ICC have provided evidence that women of parity 3 or more were significantly more likely to have pre-cancer compared to nulliparous women [34]. In Nigeria, according to the Nigeria Demographic Health Survey (NDHS) 2013, the national average number of births per woman is 5.5 [35]. In many settings in sub-Saharan Africa and Nigeria, women place a high premium on parity, and this socio-cultural norm amidst poor coverage for CCS services could contribute to the burden of pre-cancer and ICC [36]. Other sociodemographic characteristics such as smoking, sexually transmitted infections, life-time number of sexual partners, and age at first sexual intercourse have been identified as significant cofactors in cervical carcinogenesis [37]. These identified cofactors associated with abnormal cervical cytology outcomes at CCS further provide justification for the prioritization of CCS services targeting women with these identified characteristics, particularly in settings where resources are limited.

We also found that women who were referred by providers for CCS were 1.34 times more likely to have severe cervical dysplasia outcome compared to women who self-referred for CCS. The plausibility of this finding is not fully understood though it may be related to the role of providers in identifying women with risk factors for cervical cancer, or observing cervical abnormalities or presentation with symptoms, and offering selective referral for screening in this population. Related to this, we have previously reported that women who received provider referral were more likely to be older and have known risk factors for cervical cancer [22]. Additionally, more educated women were more likely to utilize available CCS services at relatively younger age [22].

Our analysis further confirms the role of women’s education in improving CCS utilization and outcomes. We found that completing at least 7–12 years of education significantly reduces the odds for severe cervical dysplasia by 25 to 35% compared to women who had fewer years of completed education. These findings are supported by previous studies showing the positive impact of educating women in improving cervical cancer outcomes [38, 39]. For instance, cervical cancer incidence and mortality are correlated with the socio-demographic index (SDI) of the population, with high SDI countries having a significantly lower ICC burden compared to low SDI countries [40]. In brief, the SDI ranges between 0 and 1 and is a summary indicator derived from measures of income per capita, educational attainment, and fertility [40]. An SDI of 1 represents a location with the highest observed educational attainment, the highest log income per capita, and the lowest fertility rate [40]. A previous related index, the human development index (HDI), which includes adult literacy rate and primary to tertiary education enrollment rates, has been shown to correlate inversely with incidence and mortality from ICC, with greater reductions in cervical cancer incidence in very high HDI compared to low HDI countries [41]. In Nigeria there is a wide regional disparity in median years of educational attainment, higher in the south-western states compared to the far north-east and north-western states [35]. The median years of educational attainment in the study area according to the NDHS 2013 report is 2.9 years [35]. The median years of educational attainment in our analytic sample was 13 years suggesting that only the more educated women utilized the available opportunistic CCS services and majority of the less educated in the larger population are either not aware or not able to overcome barriers to access the service. Our study therefore suggests the need for investment in developing and improving the educational status of women in our population as a social capital investment to improve cervical cancer outcomes. Added to this is the need for more cervical cancer education in low literacy communities to improve screening utilization [42].

The strength of our study findings is related to the relatively large sample size covering a 10-year period in an opportunistic CCS in a cosmopolitan Nigerian city that also offers care to a large population of HIV infected adults in West Africa. To the best of our knowledge this is the first secondary analysis of CCS data in Nigeria that provides precise estimates of the age at CCS and the epidemiological factors associated with an underlying abnormal cervical cytology outcome. Because the women included in this analysis were self-selected having overcome barriers to accessing opportunistic CCS services, and may not be representative of the general population of women in Nigeria or West Africa, our findings are of limited external validity to other settings in Nigeria or West Africa amongst women with ongoing HIV care and amongst areas that have availability of opportunistic cervical cancer services. Also, self-reported risk factors such as age, age at first sex, life-time number of sex partners, use of condoms and HIV status is a limitation in this analysis. It is possible that some women may have concealed their HIV status and other sensitive socio-demographic information, and this could affect the internal validity of our estimates.

In conclusion, cervical cancer is a preventable cancer and organized CCS programs such as those in the industrialized nations have dramatically reduced incidence and mortality. Future research should include a focus on understanding provider and patient perspectives on the facilitators and barriers to CCS in an opportunistic screening setting using qualitative research methodology. However, our current findings could guide health policy leaders in the implementation of CCS guidelines particularly in our settings where CCS are largely opportunistic. Specifically, our findings of a relatively late age at first cervical cancer screening particularly in HIV infected women population will require a more focused effort and investment in awareness campaigns and cervical cancer education with emphasis on the benefits of starting screening at a younger age in order to maximize the overall gains of CCS as secondary preventive service for early detection and treatment of precancerous conditions. This remain an effective health service intervention for prevention of morbidity and mortality due to ICC in the population.

## Supplementary information

**Additional file 1.** Study sample derivation for study aims 1, 2 and 3. Note: the results presented in this manuscript are from aim 2 and primary aim 3.

**Additional file 2.** The operational definition of independent variables and the primary outcome.

## Data Availability

All the relevant data for this analysis have been presented in the body of this manuscript. Additional information on the analytic sample have been provided in Additional files attached to this manuscript. The original data sources and the dataset used in this analysis is available upon reasonable request to the corresponding author.
