# Supplementary material for: HIV status, age at cervical Cancer screening and cervical cytology outcomes in an opportunistic screening setting in Nigeria: a 10-year Cross sectional data analysis
Source: Infect Agent Cancer. 2019 Nov 29;14:43. doi: 10.1186/s13027-019-0263-4 (PMC6884842; doi:10.1186/s13027-019-0263-4)
Supplement: Supplementary file 1 — Additional file 1. Study sample derivation for study aims 1, 2 and 3. Note: the results presented in this manuscript are from aim 2 and primary aim 3. [file 13027_2019_263_MOESM1_ESM.docx]

**Additional file 1**. Study sample derivation for study aims 1, 2 and 3. Note: the results presented in this manuscript are from aim 2 and primary aim 3.
