# Supplementary material for: HIV status, age at cervical Cancer screening and cervical cytology outcomes in an opportunistic screening setting in Nigeria: a 10-year Cross sectional data analysis
Source: Infect Agent Cancer. 2019 Nov 29;14:43. doi: 10.1186/s13027-019-0263-4 (PMC6884842; doi:10.1186/s13027-019-0263-4)
Supplement: Supplementary file 2 — Additional file 2. The operational definition of independent variables and the primary outcome. [file 13027_2019_263_MOESM2_ESM.docx]

**Appendix 1.** The operational definition of independent variables and the primary outcome variables

**Age at first cervical cancer screening**: This is the reported age in years at the time of first cervical cancer screening.

**Age at first sexual intercourse**: This is the reported age in years at which a woman had first penetrative intercourse.

**Total number of life time sexual partners**: This is the reported number of total life time sexual partners at the time of first cervical cancer screening.

**HIV status**: This is the reported HIV status of the woman at the time of first cervical cancer screening. This variable is captured as either “HIV infected”, “HIV uninfected”, or “HIV unknown”.

**History of vaginal infection**: This is the reported history of vaginal infection. This variable is captured as “yes”, “no”, or “unknown”.

**Ever diagnosed with a sexually transmissible infection (STI):** This is the reported history of ever receiving a diagnosis of an STI from a health care provider. This variable is captured as “yes” or “no” or “unknown”. Those who responded “yes” were asked to specify the type of STI diagnosis e.g., genital warts, syphilis, gonorrhea, etc.

**Use of condoms**: This is the reported use of condoms during sex. This variable is captured as “yes”, “no”, or “unknown”. There was no specification on frequency, or consistency of use of condoms.

**History of smoking**: This is the reported history of ever smoking up to 100 cigarettes or more. This variable is captured as “yes”, “no”, or “unknown”.

**History of alcohol consumption**: This is the reported history of alcohol consumption. This variable is captured as either “yes”, “no”, or “unknown”. It did not specify the quantity of alcohol used.

**Education years completed**: This is the reported total number of years of formal education completed.

**Parity**: This is the reported total number of deliveries that a woman has had irrespective of whether those children were alive or not at the time of first cervical cancer screening.

**Annual household income**. This is the reported total estimate of household income in the family. This estimate was provided in Nigerian naira and converted to USD for international comparison at the 2016 exchange rate of 200 naira to 1 USD (0.005).

**Source of referral**: This is the reported source of referral at first CCS. This variable is either “yes” for provider-referral or “no” for self-referral.

**Cytology outcome**: This is the cytopathological report of the first cervical cancer screening. The cytopathological interpretation and reporting was as described by the 2001 Bethesda system of reporting. The details of this classification is provided in Box 2 in Chapter 5 of this dissertation.
